# Phagocytic aberrations in macrophages in asthma: a mechanistic systematic review integrating *in vitro*, animal, and human evidence

**DOI:** 10.3389/fimmu.2026.1844956

**Published:** 2026-06-16

**Authors:** Shiyao Bai, Yue Gao, Zhiyuan Sun, Shuang Feng, Shuqi Cao, Xinming Su

**Affiliations:** Department of Respiratory and Critical Care Medicine, The First Hospital of China Medical University, Shenyang, China

**Keywords:** asthma, efferocytosis, macrophages, molecular mechanisms, phagocytosis, systematic review

## Abstract

**Objective:**

To integrate evidence from human, animal, and *in vitro* studies to elucidate the molecular mechanisms underlying phagocytic aberrations in asthmatic macrophages and to construct a cross-hierarchical mechanistic framework.

**Methods:**

This systematic review followed the PRISMA 2020 statement. PubMed and Web of Science Core Collection were searched from inception to March 6, 2026. We included original human, animal, and cellular studies on asthma that investigated macrophage phagocytic function and reported related molecular mechanisms. Two independent reviewers performed screening, data extraction, and quality assessment. Due to substantial heterogeneity across studies, a narrative synthesis approach was used to integrate the evidence.

**Results:**

A total of 37 studies, published between 1982 and 2025, were ultimately included. Overall, macrophage phagocytic function in asthma is characterized by aberrations dependent on clinical phenotype, phagocytic substrate, and the microenvironment. Based on the included studies, the relevant mechanisms were categorized into eight categories: dysregulation of phagocytic receptor signaling; defects at various stages of efferocytosis; immunometabolic reprogramming; aberrant signal transduction; regulation by immunomodulatory factors; acquired alterations in cellular function; circadian clock regulation; and other unclassified mechanisms.

**Conclusion:**

Phagocytic aberrations in macrophages in asthma represent a complex process driven by a multi-layered, interconnected molecular network. These aberrations contribute to the persistence of chronic airway inflammation, increased susceptibility to infection, elevated risk of acute exacerbations, and the development of severe or refractory asthma. Systematic integration of these mechanisms enhances the understanding of innate immune dysfunction in asthma and provides a theoretical basis for developing therapeutic strategies targeting macrophage phagocytic function.

**Systematic review registration:**

https://www.crd.york.ac.uk/prospero/, identifier CRD420261332569.

## Graphical Abstract

**Figure:**
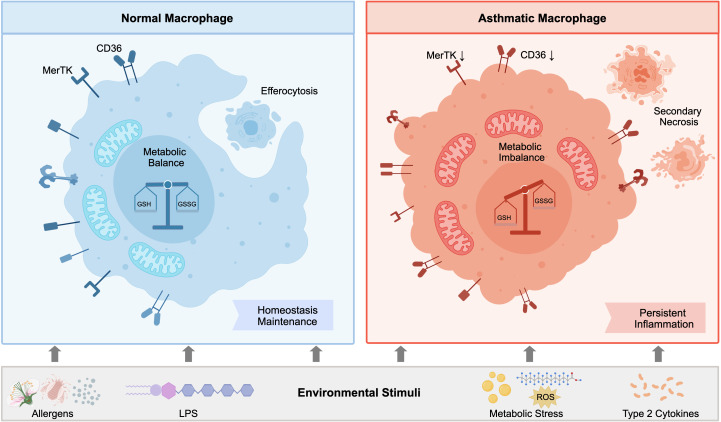
Schematic comparison of normal versus aberrant macrophage phagocytic and efferocytic function in asthma. GSH, glutathione; GSSG, oxidized glutathione; LPS, lipopolysaccharide; ROS, reactive oxygen species.

## 1 Introduction

Asthma is one of the most common chronic non-communicable diseases worldwide, affecting more than 300 million individuals across all age groups. Its prevalence continues to rise, imposing a substantial burden on global public health systems (1). Although anti-inflammatory therapy based on inhaled corticosteroids (ICS) can effectively control symptoms and reduce acute exacerbations in most patients, approximately 5%–10% of patients present with severe asthma, characterized by a suboptimal response to standard treatments or corticosteroid resistance. This underscores the need for further investigation of novel pathogenic mechanisms and therapeutic targets (2, 3).

Asthma is a highly heterogeneous chronic airway disease. Its primary pathophysiological features include chronic airway inflammation, variable airflow limitation, airway hyperresponsiveness, and airway remodeling (4). Traditionally, asthma has been considered an eosinophilic inflammatory disease primarily driven by type 2 T helper (Th2) cells, involving the coordinated action of cytokines such as interleukin (IL)-4, IL-5, and IL-13, along with various inflammatory cells (5, 6). However, with advancing understanding of asthma heterogeneity, non-eosinophilic phenotypes, characterized predominantly by neutrophil infiltration or a relative paucity of airway inflammatory cells, have received increasing attention (7). Studies have demonstrated that airway inflammation in patients with severe asthma and obesity-related asthma is predominantly neutrophilic and responds poorly to corticosteroid treatment. These findings suggest that, in addition to adaptive immune dysregulation, innate immune imbalance plays a critical role in the pathogenesis of asthma (8–10).

Macrophages are pivotal effector cells of the pulmonary innate immune system and play a central role in the pathogenesis of asthma (11). As the first cellular line of defense against inhaled pathogens and particulate matter, macrophages not only participate in innate immune responses but also regulate local inflammatory reactions and modulate adaptive immune responses by secreting various cytokines, chemoattractants, and lipid mediators, thereby contributing to the maintenance of airway microenvironment homeostasis and the amplification of inflammation (1, 12). In response to distinct local microenvironmental signals, macrophages can adopt distinct functional states. Traditionally, macrophages have been broadly categorized into classically activated pro-inflammatory phenotypes and alternatively activated pro-resolving and repair-associated phenotypes (often referred to in the literature as M1-like and M2-like, respectively) (13, 14). However, recent studies have revealed considerable heterogeneity in pulmonary macrophage populations. Alveolar macrophages (AMs), which reside on the airway surface and are readily accessible via bronchoalveolar lavage, originate primarily from fetal progenitors and maintain themselves through local self-renewal. Interstitial macrophages (IMs) reside in the lung parenchyma and are derived from both fetal precursors and circulating monocytes. While AMs primarily maintain alveolar homeostasis and immune tolerance, IMs are more actively involved in antigen presentation and inflammatory amplification (11, 15). Under asthmatic conditions, the functional boundaries between these subsets are dynamically reshaped by disease endotypes and the local microenvironment.

In allergic asthma, type 2 cytokines such as IL-4 and IL-13 can drive pulmonary macrophages toward type 2 inflammation-associated functional responses (15–17). Although this process contributes to post-inflammatory tissue repair to some extent, its sustained or excessive activation is also associated with increased mucus secretion, airway remodeling, and elevated disease severity (18, 19). Notably, the development and progression of asthma depend not only on the initiation of inflammation but also on whether inflammatory responses can be resolved and cleared in a timely and effective manner. Inflammation resolution is not a passive process but a highly regulated, active biological program (20). In this process, macrophages, particularly AMs residing in the airways and alveolar spaces, play a central role (21). On one hand, macrophages clear exogenous substances such as bacteria, viruses, fungi, and environmental particles through phagocytosis (22); on the other hand, they recognize and clear apoptotic cells, particularly eosinophils and neutrophils that accumulate in large numbers during airway inflammation, through efferocytosis, thereby promoting inflammation resolution and tissue repair (23, 24). This process is essential for preventing secondary necrosis of apoptotic cells, limiting the release of damage-associated molecular patterns (DAMPs), and maintaining local immune tolerance (25).

Accumulating evidence indicates that functional imbalance in macrophages represents a key pathogenic mechanism in asthma, particularly in severe asthma and non-eosinophilic asthma (3). Among these functional abnormalities, phagocytic defects are particularly prominent, manifesting primarily as reduced recognition, uptake, and clearance of pathogens and apoptotic cells, coupled with aberrant responses to local microenvironmental signals. This abnormality is not confined to a single process but involves functional disturbances across multiple levels, including pathogen phagocytosis, efferocytosis, and inflammation resolution, and may be closely associated with specific clinical phenotypes of asthma (23, 24). For instance, macrophages derived from children with severe asthma exhibit significantly diminished phagocytic capacity against Staphylococcus aureus and Haemophilus influenzae; similarly, macrophages from adult patients with severe asthma demonstrate markedly impaired phagocytic function against these bacteria (26). In patients with non-eosinophilic asthma, macrophage efferocytosis is impaired, which may be a significant contributor to the persistent accumulation of airway neutrophils and the chronicity of inflammation (23). Furthermore, macrophages from patients with obesity-related asthma demonstrate significantly reduced efferocytic capacity, which is associated with decreased sensitivity to glucocorticoids (22). These phagocytic defects may not only lead to inadequate clearance of inhaled pathogens and particulate matter, thereby increasing the risk of respiratory infections, but may also trigger the release of endogenous danger signals due to the accumulation of apoptotic cells and their secondary necrosis, further amplifying and prolonging airway inflammatory responses.

Although existing investigations encompassing *in vitro* experiments, animal models, and clinical observations have consistently demonstrated the presence of phagocytic aberrations in asthmatic macrophages, the underlying molecular basis requires systematic integration and comprehensive elucidation. Available evidence indicates that these functional aberrations involve multi-layered molecular regulatory networks, encompassing mechanisms such as dysregulated phagocytic receptor expression, perturbations in intracellular signal transduction cascades, and immunometabolic reprogramming (17, 24, 25, 27–29). Accordingly, this review aims to systematically synthesize evidence from *in vitro* cellular studies, animal models, and human investigations to comprehensively elucidate the molecular mechanisms underlying phagocytic aberrations in macrophages in asthma, construct a cross-hierarchical mechanistic framework, and further explore implications for disease phenotyping, clinical prognosis, and potential therapeutic interventions. The ultimate objective is to provide a theoretical foundation for advancing the understanding of immunopathological processes in asthma and for developing therapeutic strategies targeting macrophage phagocytic function.

## 2 Materials and methods

This systematic review was conducted and reported in accordance with the Preferred Reporting Items for Systematic Reviews and Meta-Analyses (PRISMA) 2020 statement (30). The review protocol was prospectively registered in PROSPERO (registration number: CRD420261332569).

### 2.1 Literature search strategy

We systematically searched the PubMed and Web of Science Core Collection databases from their inception dates to March 6, 2026, without imposing any year-of-publication restrictions. The search strategy combined Medical Subject Headings (MeSH) terms with free-text keywords, structured around the following three core concepts:

1. Asthma (e.g., asthma, bronchial hyperreactivity, airway inflammation);
2. Macrophages (e.g., macrophage, alveolar macrophage, monocyte-derived macrophage);
3. Phagocytic function and molecular mechanisms (e.g., phagocytosis, efferocytosis, phagocytic index, phagocytic function, cell clearance, and terms related to phagocytosis-associated molecules and mechanisms such as CD36, MerTK, MFG-E8, Gas6, scavenger receptor, molecular mechanism, and immunometabolism).

### 2.2 Literature screening and data extraction

**Figure 1:**
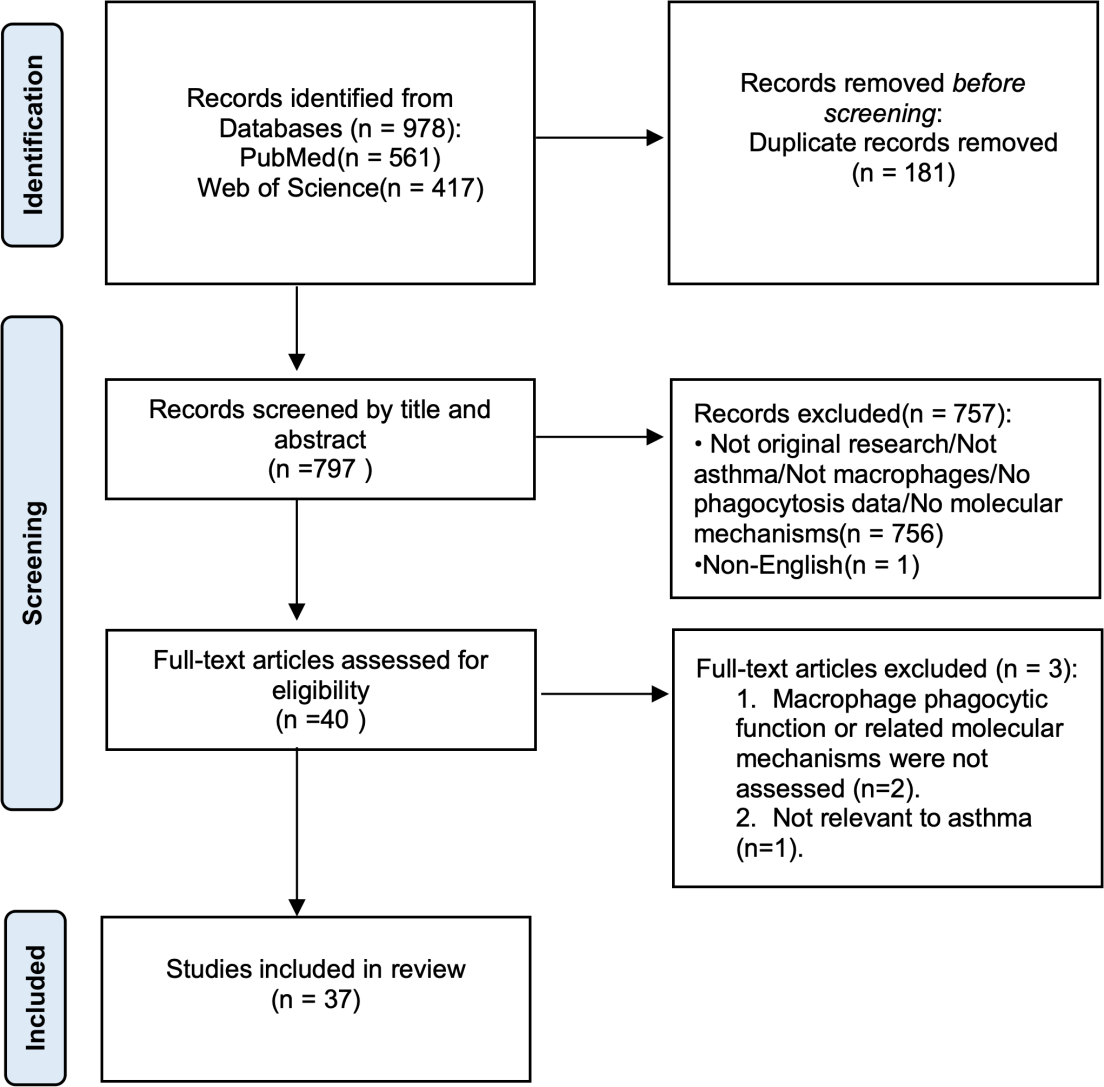
PRISMA flow diagram for the systematic review.

A total of 978 records were identified through database searches (PubMed: 561 records; Web of Science Core Collection: 417 records). Following removal of duplicate records using Rayyan software, 797 records remained and were subjected to title and abstract screening. Two independent reviewers (SB and YG) performed screening based on pre-specified inclusion and exclusion criteria. Studies were included if they were English-language original articles describing molecular mechanisms underlying macrophage phagocytic function in asthma. Studies were excluded if they did not pertain to asthma, did not involve macrophages, did not assess macrophage phagocytic function or investigate associated molecular mechanisms, or were not original research articles. After screening of titles and abstracts, 757 records were excluded, and 40 full-text articles were assessed for eligibility. Of these, two articles were excluded for failure to evaluate macrophage phagocytic function or related molecular mechanisms, and one article was excluded for not addressing asthma. Ultimately, 37 studies met the inclusion criteria and were included in the qualitative synthesis. The PRISMA flow diagram is presented in **Figure 1**.

The two reviewers independently extracted data from the included articles. The following data were extracted: study characteristics (first author, publication year, country or region, and study type); study-specific details for human, animal, and cellular studies (asthma clinical characteristics, asthma model establishment method, cell type and source, stimulation conditions or interventions, macrophage source, method for assessing phagocytic function, primary outcome measures, and key findings); and mechanism-related information pertaining to phagocytic function, including phagocytic receptors, bridging molecules, signaling pathways, and metabolic pathways. Given that the included studies comprised clinical studies, animal experiments, and *in vitro* cellular studies, corresponding quality assessment tools were applied to each study type. Specifically, the Newcastle-Ottawa Scale (NOS) was used for quality assessment of clinical studies; the SYRCLE risk of bias tool was applied to evaluate risk of bias in animal studies; and for *in vitro* cellular studies, methodological quality was assessed using a modified ToxRTool supplemented with study-specific criteria, including biological relevance of stimuli, cell viability assessment, standardization of phagocytosis assays, and the requirement for at least three independent experiments. In cases of disagreement between the two reviewers regarding extracted data or quality assessment judgments, consensus was reached through verification against the original articles and discussion; when disagreements persisted, a third reviewer (ZS) was consulted to resolve discrepancies.

### 2.3 Evidence synthesis and mechanism categorization

Given the substantial heterogeneity among the included studies with respect to study subjects (human, animal, and cellular), experimental models, stimulating factors, outcome definitions, and detection methods, a structured narrative synthesis approach was employed to integrate the evidence. Based on the findings of the included studies, prior literature, and the objectives of this review, the mechanisms underlying phagocytic aberrations in macrophages were classified into the following eight categories: dysregulation of phagocytic receptor signaling; defects at various stages of efferocytosis; immunometabolic reprogramming; aberrant signal transduction; regulation by immunomodulatory factors; acquired alterations in cellular function; circadian clock regulation; and unclassified mechanisms. Furthermore, in this review, phagocytic aberrations in macrophages broadly encompass diminished capacity for the uptake and clearance of pathogens, particulate matter, apoptotic cells, or cellular debris, with particular emphasis on impaired efferocytosis. Studies reporting abnormalities in phagocytosis-related receptors, bridging molecules, signaling pathways, or metabolic states without directly providing functional phagocytic assay data were included in the analysis only when the authors explicitly established a connection to macrophage phagocytic function and the findings provided mechanistic interpretive value.

## 3 Results

### 3.1 Literature screening results and general characteristics of included studies

This review ultimately included 37 studies, with publication years ranging from 1982 to 2025. The studies originated from 13 countries and regions across North America, Europe, Asia, Oceania, and South America, indicating that phagocytic aberrations in macrophages in asthma have become a widely and persistently investigated topic. By study type, the included studies comprised 15 human studies, 20 animal studies, and 23 cellular studies; of these, 20 studies involved two or more evidence hierarchies, such that the sum across study types exceeded the total number of included studies. Overall, the available evidence indicates that phagocytic aberrations in macrophages in asthma do not manifest simply as a global decline in phagocytic capacity, but rather exhibit distinct clinical phenotype dependence, phagocytic substrate dependence, and evidence hierarchy correlation, thereby reflecting the considerable biological complexity of this process. Regarding methodological quality, human studies were assessed using the Newcastle-Ottawa Scale (NOS), with scores ≥6 (out of 9) considered as high quality. Most included human studies met this threshold, with clear definitions of asthma phenotypes and appropriate outcome assessments, and were therefore judged to be generally of high quality. Animal studies exhibited an overall high risk of bias, particularly in domains related to selection bias (lack of randomization), performance bias (lack of blinding), and detection bias (unclear outcome assessment). A minimum risk of bias was identified in attrition and reporting domains, although incomplete reporting limited full assessment. Cellular studies showed substantial variability in methodological quality, encompassing both high-quality investigations characterized by direct mechanistic evidence and comprehensive experimental designs, and studies providing only preliminary suggestive information. Therefore, despite the accumulation of substantial mechanistic evidence pertaining to phagocytic aberrations in macrophages in asthma, comparability and consistency across different tiers of evidence remain limited. The general characteristics of the included studies are summarized in **Table 1** (24, 31–66).

**Table 1:** General characteristics of included studies.

Study ID	First author/year	Country/region	Study type	Key points	Quality assessment
001	Thomas Naessens/2012 (31)	Belgium	Animal study	Animal experiments suggest that alveolar macrophage phagocytic function is reduced in the asthmatic state, with exploration of underlying mechanisms.	Some concerns
002	L. Mahajan/2008 (32)	India	Cellular study	Co-culture of mouse macrophage cell line with apoptotic eosinophils from asthma patients to assess macrophage phagocytic function and regulatory mechanisms.	High quality
003	Sangjoon Lee/2021 (33)	Japan	Comprehensive (animal + cellular)	Animal experiments evaluate asthmatic inflammatory changes; cellular studies assess macrophage phagocytic function and validate related mechanisms.	Animal: high risk of bias; Cellular: high quality
004	Susumu Isogai/2007 (34)	Canada	Comprehensive (animal + cellular)	Animal experiments observe asthma-related changes in macrophage phagocytosis; cellular studies further elucidate IFN-γ-related mechanisms.	Animal: high risk of bias; Cellular: moderate quality
005	Philippe Godard/1982 (35)	France	Human study	Alveolar macrophages derived from BALF of asthma patients and healthy controls were used to compare phagocytic capacity and release of phagocytosis-related mediators.	NOS 5/9
006	M. Damon/1990 (36)	France	Human study	Comparison of activation status and functional products related to phagocytosis in alveolar macrophages from asthma patients and healthy controls.	NOS 5/9
007	Xudong Zhang, Mingqiang Zhang/2021 (37)	China	Comprehensive (animal + cellular)	Cellular studies reveal mechanistic basis of macrophage phagocytic/uptake function; animal experiments validate functional consequences in asthmatic inflammation	Animal: high risk of bias; Cellular: low quality
008	Jia Wang/2014 (38)	China	Comprehensive (animal + cellular)	In vivo–in vitro linked design: animal experiments observe indirect phenotypic changes, with mechanistic validation using macrophages isolated from the same model.	Animal: high risk of bias; Cellular: low quality
009	Jianan Wan/2019 (39)	China	Comprehensive (animal + cellular)	Animal experiments reveal impaired phagocytic function in asthma; cellular studies further elucidate related molecular regulatory mechanisms	Animal: high risk of bias; Cellular: moderate quality
010	Julia Teppan/2024 (40)	Austria	Comprehensive (human + animal + cellular)	Human studies reveal circadian clock disruption in macrophages; cellular studies elucidate molecular mechanisms; validation in animal models	Human: NOS 5/9; Animal: high risk of bias; Cellular: moderate quality
011	Jong Huat Tee/2023 (41)	Singapore	Comprehensive (animal + cellular)	Public database analysis identifies associations of reduced ISM1 and adiponectin with asthma; functional validation through animal and cellular experiments.	Animal: high risk of bias; Cellular: moderate quality
012	J. L. Simpson/2013 (42)	Australia	Human study	Comparison of macrophage efferocytic function in asthma patients with different inflammatory phenotypes.	NOS 6/9
013	Reinaldo C. Silva/2010 (43)	Brazil	Comprehensive (animal + cellular)	Animal and cellular studies together construct mechanistic pathway through which inflammatory mediators influence macrophage function.	Animal: high risk of bias; Cellular: moderate quality
014	Alexandre P. Rogerio /2012 (44)	U.S.	Comprehensive (animal + cellular)	Cellular studies elucidate mechanisms of pro-resolving lipid mediators; animal studies provide in vivo validation.	Animal: high risk of bias; Cellular: high quality
015	Haruka Miki/2021 (45)	U.S.	Comprehensive (animal + cellular)	Animal experiments identify efferocytosis-related phenomena; cellular studies reveal mechanisms; causal relationship validated through adoptive transfer.	Animal: high risk of bias; Cellular: moderate quality
016	Hiroto Matsuse/2013 (46)	Japan	Animal study	Animal experiments evaluate macrophage-related functional changes in the context of asthmatic airway inflammation.	High risk of bias
017	Xingyue Liu/2025 (24)	China	Comprehensive (animal + cellular)	Animal and cellular studies together elucidate regulatory mechanisms of phagocytic receptors in macrophages in asthma.	Animal: high risk of bias; Cellular: moderate quality
018	Zhike Liang/2014 (47)	U.K.	Human study	Comparison of bacterial phagocytic capacity of macrophages from patients with severe versus non-severe asthma; analysis of association with lung function and inflammatory parameters.	NOS 7/9
019	J. C. Lay/2009 (48)	U.S.	Human study	Dual in vivo and in vitro validation strategy to assess macrophage phagocytic function in patients with mild asthma.	NOS 7/9
020	Neeta Kulkarni/2018 (49)	U.K.	Human study	Self-controlled before–after study evaluating the effect of allergen avoidance on macrophage phagocytic function in asthmatic. children.	NOS 6/9
021	Mai-Lan N. Huynh/2005 (50)	U.S.	Comprehensive (human + cellular)	Clinical observation reveals reduced macrophage phagocytic function in severe asthma; in vitro experiments explore related mechanisms.	Human: NOS 9/9; Cellular: moderate quality
022	Peikai Huang, Shushan Wei/2019 (51)	China	Comprehensive (animal + cellular)	In vivo intervention–in vitro detection design to analyze phagocytosis-related changes in macrophages under different treatment conditions.	Animal: high risk of bias; Cellular: moderate quality
023	Huiling Hong/2024 (52)	China	Comprehensive (animal + cellular)	Phenotype validation through animal experiments; combined with cellular studies to explore circadian clock-related mechanisms.	Animal: high risk of bias; Cellular: moderate quality
024	Jocelyn R. Grunwell/2019 (53)	U.S.	Human study	Analysis of alveolar macrophage phagocytic function and pro-inflammatory characteristics in children with severe asthma stratified by neutrophilic inflammatory phenotype.	NOS 5/9
025	Marianne Geiser/2014 (54)	Switzerland	Cellular study	Primary macrophages isolated from asthmatic mice subjected to in vitro interventions to evaluate particle uptake behavior.	Moderate quality
026	Marianne Geiser/2013 (55)	U.S.	Cellular study	Primary macrophages derived from asthma patients used for in vitro interventions to analyze changes in phagocytic receptors and internalization function.	Moderate quality
027	Anne M. Fitzpatrick/2011 (56)	U.S.	Comprehensive (human + cellular)	Alveolar macrophages from asthma patients collected for direct phagocytic function assessment, with further mechanistic studies.	Human: NOS 6/9; Cellular: moderate quality
028	Anne M. Fitzpatrick/2008 (57)	U.S.	Comprehensive (human + cellular)	Alveolar macrophages from asthma patients collected for direct phagocytic function assessment, followed by in vitro intervention studies.	NOS 7/9; Cellular: low quality
029	Ruby Fernandez-Boyanapalli, et al /2013 (58)	U.S.	Human study	Investigation of efferocytosis abnormalities in obesity-related asthma and associated metabolic mechanisms.	NOS 7/9
030	Jennifer M. Felton, Christopher D. Lucas/2018 (59)	U.K.	Comprehensive (animal + cellular)	Animal and cellular experiments to validate the role of MerTK-related pathways in phagocytic function.	Animal: high risk of bias; Cellular: moderate quality
031	Eric Careau/2006 (60)	Canada	Animal study	Animal experiments evaluate functional changes in pulmonary macrophages under inflammatory conditions.	High risk of bias
032	Rossa E Brugha/2014 (61)	U.K.	Comprehensive (human + cellular)	Clinical observation identifies phagocytic defects; cellular experiments elucidate PGE_2_-related mechanisms.	Human: NOS 7/9; Cellular: moderate quality
033	Faouzi Braza,Stephanie Driou,Virginie Forest/2016 (62)	France	Animal study	Evaluation of the interaction between mesenchymal stromal cells and pulmonary macrophages in asthmatic inflammation.	High risk of bias
034	Claudiney de Freitas Alves/2013 (63)	Brazil	Comprehensive (animal + cellular)	Animal experiments evaluate airway inflammation improvement; cellular studies analyze the effect of ellagic acid on macrophage phagocytic function.	Animal: high risk of bias; Cellular: high quality
035	Neil E. Alexis/2001 (64)	U.S.	Human study	Assessment of changes in phagocytic receptors on macrophages from patients with eosinophilic asthma.	NOS 7/9
036	Neil E. Alexis/2003 (65)	U.S.	Human study	Self-controlled crossover design to evaluate changes in phagocytic receptors and phagocytic function following LPS exposure.	NOS 7/9
037	Soraia Abreu/2023 (66)	Brazil	Comprehensive (animal + cellular)	Mechanistic studies using cells pre-treated with serum from asthma patients; anti-inflammatory effects further validated in animal models.	Animal: high risk of bias; Cellular: moderate quality

Study types include human studies, animal studies, cellular studies, and comprehensive studies. Comprehensive studies refer to investigations that simultaneously incorporate two or more evidence hierarchies, such as human and in vitro, animal and in vitro, or human, animal, and in vitro. Quality assessment was performed using the Newcastle-Ottawa Scale (NOS), the Systematic Review Centre for Laboratory Animal Experimentation (SYRCLE) risk of bias tool, and the modified ToxRTool, respectively, according to study type.

### 3.2 Human studies: phenotype-dependent phagocytic aberrations in macrophages in asthma

The 15 included human studies exhibited a multi-layered integrative design, comprising 10 studies restricted to human subjects, 4 human-cellular combined studies, and 1 study that simultaneously integrated evidence from human, animal, and cellular levels. The sample sizes of asthma patient groups across these studies ranged from 10 to 80 participants, while those of healthy control groups ranged from 4 to 47 participants. Some studies did not include healthy controls but instead employed comparisons between different asthma subgroups or patient self-controlled designs. The study populations encompassed diverse clinical phenotypes, including mild to severe asthma, allergic and non-allergic asthma, and eosinophilic and non-eosinophilic asthma. Macrophages were primarily derived from bronchoalveolar lavage fluid (BALF) (8 studies), induced sputum (7 studies), and peripheral blood monocyte-derived macrophages (4 studies). Primary outcome measures included phagocytic percentage or phagocytic index, efferocytic efficiency, expression of phagocytic receptors, and levels of related inflammatory mediators or metabolites. Overall, human studies indicate that phagocytic aberrations in macrophages from asthma patients are closely associated with disease severity, inflammatory phenotype, age, treatment background, and environmental exposure. The general characteristics of the included human studies are summarized in **Table 2**.

**Table 2:** General characteristics and major findings of included human studies.

Study ID	Study population and sample size	Macrophage source	Primary outcome measures	Key findings
005	Allergic asthma (n = 15), aspirin-sensitive asthma (n = 6), healthy controls (n = 7)	BALF	Phagocytic percentage, release of phagocytosis-related mediators	Phagocytic capacity of alveolar macrophages from asthma patients significantly reduced; release of PGE_2_, PGF_2_α, and TxB_2_ decreased following phagocytic stimulation. These changes negatively correlated with eosinophil proportion in BALF.
006	Asthma (n = 12), healthy controls (n = 5) for phagocytosis-related product detection; asthma (n = 8), healthy controls (n = 9) for receptor-mediated cell activation function detection	BALF	Phagocytosis-related product detection, receptor-mediated cell activation function detection	Phagocytosis-related reactive oxygen species production increased in alveolar macrophages from asthma patients, positively correlated with disease severity; sustained activation state maintained even during asymptomatic periods.
010	Allergic asthma (n = 4), non-allergic asthma (n = 4), healthy controls (n = 4)	Peripheral blood monocytes	Circadian clock protein expression	Disrupted expression rhythms of circadian clock proteins in asthma patients.
012	Eosinophilic asthma (n = 20), non-eosinophilic asthma (n = 30)	Induced sputum, BALF	Efferocytic efficiency	Macrophage efferocytic function significantly impaired in non-eosinophilic asthma patients. Phagocytic function showed negative correlation with ICS dosage, with age identified as an independent influencing factor.
018	Severe asthma (MDMs n = 14, AMs n = 8), non-severe asthma (MDMs n = 14, AMs n = 6), healthy controls (MDMs n = 14, AMs n = 7)	BALF, peripheral blood mononuclear cells	Standardized total fluorescence intensity	AMs and MDMs from severe asthma patients showed selective impairment in phagocytosis of Haemophilus influenzae and Staphylococcus aureus; phagocytic capacity positively correlated with FEV_1_% and negatively correlated with airway eosinophilic inflammation.
019	Mild allergic asthma (n = 10), healthy controls (n = 8)	Induced sputum	In vivo phagocytic percentage, ex vivo MFI, phagocytic cell percentage, particles per cell, CD86 and CD64 expression	Both in vivo and ex vivo phagocytic capacity enhanced in asthma patients, closely correlated with macrophage proportion in sputum; upregulation of CD86 expression suggesting enhanced antigen-presenting capacity.
020	Children with mild-to-moderate asthma (GINA 1–3, n = 62), self-controlled before–after design	Induced sputum	Phagocytic index, phagocytic percentage, phagocytic intensity	Macrophage phagocytic function significantly decreased following allergen avoidance in asthmatic children.
021	Mild-to-moderate asthma (n = 25), severe asthma (n = 18, including n = 12 receiving corticosteroid pulse therapy), healthy controls (n = 14)	BALF	Phagocytic index	Phagocytic index of alveolar macrophages significantly reduced in severe asthma patients; increased after corticosteroid pulse therapy compared to pre-treatment levels.
024	Moderate asthma (n = 13), severe low-neutrophil asthma (n = 29), severe high-neutrophil asthma (n = 38)	BALF	Phagocytic index, macrophage apoptosis, inflammatory cytokines	Macrophages from children with severe high-neutrophil asthma showed impaired phagocytic function, increased apoptosis, and a pro-inflammatory phenotype.
027	Moderate asthma (n = 21), severe asthma (n = 43)	BALF	Phagocytic index, apoptosis, IL-8, HDAC activity, GSH/GSSG homeostasis	Phagocytic function of alveolar macrophages decreased in children with severe asthma, accompanied by elevated GSSG, reduced HDAC activity, enhanced inflammation, and increased apoptosis.
028	Moderate asthma (n = 14), severe asthma (n = 16), healthy controls (n = 10), chronic cough controls (n = 10)	BALF	Phagocytic index, phagocytic percentage, MFI, apoptosis, percentage of external binding	Phagocytic function of alveolar macrophages significantly decreased in children with severe asthma, accompanied by increased apoptosis and external binding.
029	Non-obese asthma (n = 19), obese asthma (n = 14), non-obese healthy controls (n = 19), obese healthy controls (n = 6)	Induced sputum, peripheral blood	Efferocytic index, metabolic parameters	Efferocytic capacity of macrophages decreased in obesity-related asthma, accompanied by metabolic dysregulation.
032	Mild asthma (n = 13), moderate-to-severe asthma (n = 36), healthy controls (n = 47)	Induced sputum	Particle phagocytosis	Phagocytic function of macrophages against particulate matter impaired in children with severe asthma, associated with airway eosinophilic inflammation and PGE_2_.
035	Mild eosinophilic asthma (n = 9), mild non-eosinophilic asthma (n = 11), healthy controls (n = 20)	Induced sputum	MFI, phagocytic receptor expression	Phagocytic function impaired in macrophages from patients with mild eosinophilic asthma, with downregulation of CD64 and CD11b expression; CD64 expression positively correlated with phagocytic function.
036	Mild allergic asthma (n = 10), self-controlled before–after LPS inhalation	Induced sputum, peripheral blood	Phagocytic receptor expression	Inhaled LPS significantly suppressed macrophage phagocytic function in asthma patients, correlated with degree of airway neutrophilic inflammation, accompanied by downregulation of CD11b expression.

BALF, bronchoalveolar lavage fluid; AMs, alveolar macrophages; MDMs, monocyte-derived macrophages; ICS, inhaled corticosteroids; MFI, mean fluorescence intensity. As some studies performed multiple functional assays using the same samples, sample sizes may be reported separately according to the specific assay.

In patients with severe asthma, the phagocytic capacity of macrophages derived from either BALF or peripheral blood monocyte-derived macrophages is significantly diminished against bacteria such as Haemophilus influenzae and Staphylococcus aureus, and this functional impairment correlates with decreased lung function and enhanced airway eosinophilic inflammation (47). Correspondingly, studies have demonstrated that following corticosteroid pulse therapy, the phagocytic index of macrophages from patients with severe asthma increased relative to pre-treatment levels, suggesting that macrophage phagocytic function may be dynamically regulated by the local inflammatory milieu and anti-inflammatory interventions (50). Furthermore, Grunwell et al. reported that patients with severe neutrophilic asthma exhibited more pronounced impairment of macrophage phagocytic function, accompanied by increased apoptosis and an enhanced pro-inflammatory phenotype (53). Similar observations have been reported in studies of pediatric patients with severe asthma, where a decreased macrophage phagocytic index was associated with increased apoptosis, elevated levels of inflammatory cytokines, and disrupted intracellular redox homeostasis (56, 57, 61). Collectively, these findings indicate that impaired macrophage phagocytic function is closely linked to persistent inflammatory states and oxidative stress in severe asthma.

In contrast to severe asthma, patients with mild allergic asthma may exhibit enhanced macrophage phagocytic capacity, accompanied by upregulated expression of the co-stimulatory molecule CD86 on the macrophage surface, suggesting that macrophages in this population exist in a relatively activated state characterized by augmented phagocytic function and antigen-presenting capacity (48). Another self-controlled study revealed that following allergen avoidance, macrophage phagocytic function paradoxically decreased, further indicating that phagocytic activity is dynamically modulated by the local microenvironment and inflammatory status (49).

Additionally, following inhaled lipopolysaccharide (LPS) challenge, patients with mild allergic asthma demonstrated downregulated expression of phagocytosis-related receptors and suppressed phagocytic function, changes that were positively correlated with the degree of airway neutrophilic inflammation (64). This finding suggests that under conditions of neutrophilic inflammation or increased exogenous inflammatory stimulation, macrophages from patients with asthma may exhibit a state of disequilibrium characterized by heightened sensitivity to specific stimuli yet impaired execution of clearance functions. Meanwhile, in studies of patients with non-eosinophilic asthma, age was identified as an independent factor associated with macrophage phagocytic function, whereby each one-year increment in age corresponded to approximately a 0.6% decrease in phagocytic capacity, and this function was negatively correlated with ICS dosage (42).

In summary, human studies indicate that phagocytic aberrations in macrophages in asthma exhibit considerable heterogeneity and phenotype dependence. Overall, severe asthma, especially the neutrophilic phenotype, tends to manifest as impaired phagocytic function, whereas mild allergic asthma may present with enhanced phagocytic capacity or altered macrophage activation states.

### 3.3 Animal and cellular studies: regulation of macrophage phagocytic function by the asthmatic inflammatory microenvironment

The 20 included animal studies primarily employed asthma models induced by ovalbumin (OVA), house dust mite (HDM), and Dermatophagoides farinae (Der f). Experimental animals were predominantly BALB/c and C57BL/6 mice, with a minority of studies utilizing BN rats and Wistar rats. Overall, animal studies support that the asthmatic inflammatory microenvironment significantly alters macrophage phagocytic function. Regarding methodological quality, animal studies exhibited an overall high risk of bias, with one study classified as presenting some concerns and 19 studies judged to be at high risk of bias. Specifically, the risk of selection bias was high due to lack of reporting on random sequence generation and allocation concealment. Performance bias and detection bias were present because blinding of investigators and outcome assessors was not reported. Attrition bias could not be ruled out due to incomplete reporting of animal exclusion or loss to follow-up. Furthermore, reporting bias was possible owing to the absence of prospective registration or sample size estimation. Finally, sex bias (a form of selection bias) was identified, as most studies used only female animals. Importantly, these biases stemmed primarily from inadequate methodological reporting rather than from inherent flaws in study design per se. Some animal studies directly measured macrophage phagocytic capacity, thereby confirming dysregulation of macrophage phagocytic function under asthmatic conditions; other studies, while not employing traditional phagocytosis assays as primary endpoints, indirectly demonstrated a close association between altered macrophage phagocytic function and asthmatic airway inflammation through manipulation of phagocytosis-related molecules, signaling pathways, or cellular phenotypes. In addition to studies that directly observe macrophage phagocytic function in asthmatic animals, we also included studies using gene-knockout mice, pharmacological inhibitors, or exogenous protein administration. Although these latter studies do not directly reflect macrophage dysfunction caused by the asthmatic state per se, they provide mechanistic insights into how specific molecular pathways regulate macrophage phagocytic function under asthmatic conditions. These studies are therefore interpreted as supporting molecular mechanisms rather than as direct evidence of asthma-induced dysfunction. The general characteristics of the included animal studies are summarized in **Table 3**.

**Table 3:** General characteristics and major findings of included animal studies.

Study ID	Species/strain	Sex/age	Asthma model	Macrophage source	Primary outcome measures	Key findings
001	C57BL/6	Female/6–8 weeks	OVA sensitization + challenge	BALF	Phagocytic cell percentage, particles per cell	Decreased proportion of phagocytic cells in BALF of asthmatic mice, with significantly reduced number of microspheres phagocytosed per positive cell.
003	C57BL/6	Not reported/8–12 weeks	OVA sensitization + challenge	BALF	Airway inflammation parameters	Arf6 deficiency alleviated airway inflammation and reduced IL-1β secretion in asthmatic mice.
004	BN	Male/7–9 weeks	OVA sensitization + challenge	BALF	Phagocytic percentage, efferocytic efficiency	Following CD8^+^ γδ T cell intervention, alveolar macrophage phagocytic clearance of eosinophils was enhanced, an effect dependent on IFN-γ.
007	C57BL/6	Not reported/8–10 weeks	HDM sensitization + challenge	BALF, lung tissue	Antigen presentation function parameters, type 2 inflammation parameters	IRAK-M deficiency led to enhanced macrophage activation and exacerbated type 2 inflammation.
008	BALB/c	Female/4 weeks	OVA sensitization + challenge, RSV-induced exacerbation	Lung tissue	Macrophage activation marker genes	Macrophage activation was enhanced in asthmatic mice; activation was suppressed following RSV-induced exacerbation, while medium and high doses of BSYQF restored activation status.
009	BALB/c	Female/6 weeks	OVA sensitization + challenge	Peripheral blood mononuclear cells, lung tissue, BALF	Rac1 expression, airway inflammation parameters	Downregulation of Rac1 and elevation of inflammatory cytokine IL-33 in asthmatic mice.
010	BALB/c	Not reported/12 weeks	HDM sensitization + challenge	BALF	Inflammatory parameters, macrophage subset counts	ROR inverse agonist reduced the number of pro-inflammatory alveolar macrophages and lung tissue inflammatory infiltration in asthmatic mice.
011	C57BL/6	Not reported/7–8 weeks	HDM sensitization + challenge	BALF	Efferocytic efficiency, inflammatory parameters	ISM1 deficiency led to impaired efferocytic function in macrophages and exacerbated inflammation in asthmatic mice.
013	Wistar	Male/8–12 weeks	OVA sensitization + challenge	BALF	Phagocytic index, bactericidal capacity	Both phagocytic and bactericidal functions of alveolar macrophages were enhanced in asthmatic rats; leukotriene receptor antagonist reversed this enhancement.
014	FVB	Male/5–7 weeks	OVA sensitization + challenge	Peritoneal macrophages, BALF	Phagocytic index	AT-RvD1 enhanced phagocytic function of macrophages in asthmatic mice.
015	C57BL/6	Male/6–8 weeks	HDM sensitization + challenge	BALF, lung tissue	Phagocytic percentage, functional validation parameters	Alveolar macrophages that had phagocytosed apoptotic cells suppressed airway inflammation in asthmatic mice.
016	BALB/c	Female/4 weeks	Der f sensitization + challenge, Aspergillus fumigatus infection exacerbation	Lung tissue	Phagocytic percentage	Phagocytic function of alveolar macrophages significantly impaired in asthmatic mice.
017	BALB/c	Female/9 weeks	OVA sensitization + challenge	BALF	Phagocytic percentage, expression of phagocytic receptors and regulatory factors	Phagocytic function of alveolar macrophages significantly impaired in asthmatic mice, with decreased CD36 expression and increased ADAM17 expression; these changes were reversed by TNF-α antibody blockade.
022	C57BL/6	Female/6–8 weeks	OVA sensitization + challenge	BALF	Airway inflammation parameters, oxidative stress parameters	Hydrogen inhalation activated the Nrf2/HO-1 antioxidant pathway, inhibited oxidative stress and type 2 inflammation, and alleviated airway inflammation in asthmatic mice.
023	C57BL/6	Male/10–12 weeks	HDM sensitization + challenge	BALF	Phagocytic percentage, airway inflammation parameters	Bmal1 deficiency enhanced alveolar macrophage phagocytic function and alleviated airway inflammation in asthmatic mice.
030	C57BL/6	Female/8–16 weeks	OVA sensitization + challenge	BALF	Phagocytic percentage, efferocytic efficiency, airway inflammation parameters	MerTK deficiency led to decreased efferocytic capacity of alveolar macrophages and exacerbated airway inflammation.
031	BN	Male/11–12 weeks	OVA sensitization + challenge	BALF	Phagocytic percentage, functional validation parameters	OVA sensitization reduced phagocytic function of alveolar macrophages in rats, with persistent effects on macrophage function.
033	BALB/c	Not reported/6–8 weeks	Der f sensitization + challenge	Lung tissue	In vivo phagocytosis tracking, macrophage polarization state, airway inflammation parameters	Following in vivo phagocytosis by pulmonary macrophages, MSCs induced polarization toward a pro-resolving and anti-inflammatory phenotype and alleviated airway inflammation in asthmatic mice.
034	BALB/c	Female/5–7 weeks	OVA sensitization + challenge	BALF	Airway inflammation parameters	Ellagic acid significantly alleviated airway inflammation in asthmatic mice.
037	C57BL/6	Female/8–10 weeks	HDM sensitization + challenge	BALF	Airway inflammation parameters, collagen deposition, lung function	Transplantation of human mesenchymal stromal cells pre-treated with serum from asthma patients alleviated airway inflammation, reduced collagen deposition, and improved lung function.

OVA, ovalbumin; HDM, house dust mite; Der f, *Dermatophagoides farinae*; BALF, bronchoalveolar lavage fluid; RSV, respiratory syncytial virus.

Animal studies further indicate that phagocytic aberrations in macrophages represent a reproducible phenomenon within the asthmatic inflammatory microenvironment. Across various asthma animal models (including BALB/c or C57BL/6 mice with OVA-induced asthma (24, 31), C57BL/6 mice with HDM-induced asthma (46), and BN rats with OVA-induced asthma (60)), the proportion of phagocytic alveolar macrophages, the phagocytic index, and the number of particles ingested per cell were observed to decrease, exhibiting trends consistent with the degree of airway inflammation. These findings suggest that the asthma-associated inflammatory milieu itself can impair the uptake capacity of alveolar macrophages (24, 31, 46, 60). Furthermore, under specific intervention conditions, enhancing macrophage phagocytic or efferocytic capacity has been shown to attenuate asthmatic airway inflammation. For instance, following CD8+ γδ T cell intervention in a rat model of OVA-induced asthma, phagocytosis of apoptotic eosinophils by rat alveolar macrophages was enhanced under interferon-gamma (IFN-γ) stimulation, accompanied by improved airway inflammation (34). In a BALB/c mouse model of OVA-induced asthma, ellagic acid was shown to enhance the phagocytic capacity of mouse alveolar macrophages toward apoptotic cells, thereby alleviating airway inflammation (63). Moreover, in a BALB/c mouse model of Der f-induced asthma, following phagocytosis of mesenchymal stem cells (MSCs) by pulmonary macrophages *in vivo*, these macrophages acquired a pro-resolving and anti-inflammatory phenotype, which subsequently attenuated airway inflammation and hyperresponsiveness in asthmatic mice (62). Abreu et al. further demonstrated that transplantation of human mesenchymal stromal cells pre-treated with serum from patients with asthma into HDM-induced asthmatic C57BL/6 mice significantly improved airway inflammation, collagen deposition, and lung function parameters (66). Beyond phenotypic observations, animal studies have also revealed potential therapeutic targets associated with macrophage phagocytosis and activation. In a murine asthma model induced by OVA sensitization and exacerbated by respiratory syncytial virus (RSV) challenge in BALB/c mice, activation of lung tissue macrophages was suppressed; however, intervention with medium and high doses of BSYQF partially restored this activation state (38). Conversely, in a C57BL/6 mouse model of HDM-induced asthma, interleukin-1 receptor-associated kinase M (IRAK-M) gene deletion resulted in enhanced activation of alveolar and lung tissue macrophages, accompanied by exacerbated type 2 inflammation (37). Overall, animal studies indicate that phagocytic aberrations in macrophages are not merely epiphenomena accompanying asthma inflammation but represent critical determinants influencing the persistence, exacerbation, and resolution of airway inflammation.

*In vitro* cellular studies provide more direct mechanistic support for the phenomena described above. The 23 included cellular studies involved primary human and mouse alveolar macrophages, human airway macrophages, human peripheral blood monocyte-derived macrophages, and various mouse murine macrophage cell lines (e.g., RAW264.7, J774A.1, MH-S, AMJ2-C8). Common asthma-relevant stimulating conditions included HDM, IL-4, IL-13, serum from patients with asthma, and apoptotic cells derived from asthmatic subjects; experimental interventions encompassed gene knockout, receptor agonists or antagonists, and pharmacological treatments. By quantifying phagocytic function parameters, including phagocytic index, mean fluorescence intensity (MFI), and phagocytic percentage, alongside mechanism-related indicators such as receptor expression, signaling pathway molecules, and metabolites, these studies have elucidated a multi-layered regulatory network underlying phagocytic aberrations in macrophages. This body of work connects alterations in the local asthmatic inflammatory microenvironment and abnormalities in key molecules to the resultant phagocytic functional phenotype, thereby forming, together with animal studies, a relatively complete experimental causal chain of evidence. The general characteristics of the included cellular studies are summarized in **Table 4**.

**Table 4:** General characteristics and major findings of included cellular studies.

Study ID	Cell type	Cell source	Asthma-relevant stimulating conditions	Intervention	Primary outcome measures	Key findings
002	J774A.1 macrophages	Mouse macrophage cell line	Apoptotic eosinophils from asthma patients	rhSP-D	Phagocytic index	rhSP-D increased macrophage uptake of apoptotic eosinophils, demonstrating enhanced efferocytosis.
003	Primary airway macrophages	C57BL/6	None	Arf6	Phagocytic percentage, IL-1β	Arf6 deficiency significantly reduced macrophage phagocytosis of ASC specks and decreased IL-1β secretion.
004	Primary alveolar macrophages	BN	None	rrIFN-γ	Phagocytic percentage	IFN-γ enhanced alveolar macrophage phagocytic function.
007	Primary alveolar macrophages/lung tissue macrophages	C57BL/6	None	IRAK-M	MFI	IRAK-M deficiency enhanced macrophage antigen uptake capacity.
008	Primary alveolar macrophages	BALB/c	OVA+RSV	BSYQF	MFI, macrophage activation marker genes	BSYQF improved macrophage activation status and enhanced phagocytic function.
009	RAW264.7 cells	Mouse macrophage cell line	None	Rac1	Phagocytic percentage, antigen presentation parameters, IL-33	Rac1 downregulation impaired macrophage phagocytic function, increased IL-33 secretion, and enhanced antigen-presenting capacity.
010	Primary monocyte-derived macrophages	Human peripheral blood	None	ROR	MFI	ROR inverse agonist enhanced macrophage phagocytic function.
011	MH-S cells	Mouse alveolar macrophage cell line	None	rISM1 and adiponectin	Efferocytic efficiency	ISM1/adiponectin enhanced macrophage clearance of apoptotic cells.
013	Primary alveolar macrophages	Wistar	None	LTC_4_	Phagocytic index	Leukotrienes directly enhanced macrophage phagocytic function.
014	AMJ2-C8 cells	Mouse alveolar macrophage cell line	None	AT-RvD1 /RvD1	Phagocytic index, macrophage metabolite detection	AT- AT-RvD1 resisted metabolic inactivation of macrophages and directly enhanced phagocytic clearance capacity.
015	Primary alveolar macrophages	C57BL/6J	HDM	ATL	ATL RALDH1, inflammatory cytokines	Following phagocytosis of apoptotic cells, macrophages upregulated RALDH1 and suppressed TNF and IL-6 production; ATL further enhanced this effect.
017	Primary alveolar macrophages	BALB/c	OVA+TNF-α	CD36, ADAM17	Phagocytic percentage, expression of phagocytic receptors and regulatory factors	TNF-α suppressed macrophage phagocytic function, upregulated ADAM17 expression, and downregulated CD36 expression; both CD36 and ADAM17 participated in phagocytic regulation.
021	Primary alveolar macrophages	Human BALF	Asthma patients	LPS	Phagocytic index, inflammatory cytokines	Macrophages from severe asthma patients showed no phagocytic response to LPS stimulation, with reduced LPS-induced inflammatory cytokine secretion.
022	Primary alveolar macrophages	C57BL/6J	OVA	Nrf2	MFI	Nrf2/HO-1 pathway involved in regulation of macrophage phagocytic function.
023	Bone marrow-derived macrophages	C57BL/6J	HDM	Bmal1	MFI, phagocytic receptor expression	Bmal1 deficiency enhanced macrophage phagocytic function and upregulated expression of phagocytosis-related receptors such as CD64 and CD11c.
025	Primary alveolar macrophages	BALB/c	OVA	Fungal spores + AuNP	Phagocytic percentage, macrophage morphology	Macrophages from asthmatic mice were activated, but phagocytosis of nanoparticles showed no significant change.
026	Primary airway macrophages	Human induced sputum	Patients with mild HDM allergic asthma	γ-Tocopherol	Phagocytic percentage, internalization/attachment ratio, phagocytic receptors, antigen presentation-related molecules	γ-Tocopherol downregulated expression of CD206, CD36, CD16, CD86, and CD40, and selectively impaired internalization capacity of opsonized particles.
027	Primary alveolar macrophages	Human BALF	Moderate-to-severe asthma patients	GSH	Phagocytic index, macrophage apoptosis, GSSG	Exogenous GSH supplementation reduced intracellular GSSG levels, restored phagocytic function, and inhibited apoptosis in alveolar macrophages from children with severe asthma.
028	Primary alveolar macrophages	Human BALF	Moderate-to-severe asthma patients	LPS	Phagocytic index, phagocytic cell percentage, MFI, apoptosis	LPS stimulation further impaired phagocytic function of alveolar macrophages in children with asthma, particularly severe cases, accompanied by increased apoptosis.
030	Primary alveolar/bone marrow-derived macrophages	C57BL/6	None	MerTK	Phagocytic percentage	MerTK deficiency led to reduced phagocytic capacity of macrophages.
032	Primary macrophages	Human peripheral blood, rat BALF	PM_10_	PGE_2_, PGD_2_	Particle phagocytosis	PGE_2_, but not PGD_2_, inhibited phagocytosis of urban PM_10_ particles by both human and rat macrophages.
034	Primary alveolar macrophages	BALB/c	OVA	Ellagic acid	Phagocytic index	Low concentration (1 μM) of ellagic acid enhanced phagocytic function of alveolar macrophages in asthmatic mice, while higher concentrations showed no significantpromoting effect.
037	Primary alveolar macrophages	C57BL/6	Pre-treatment with serum from asthma patients	hMSC	Phagocytic percentage, macrophage polarization state	Pre-treatment with serum from asthma patients rendered hMSCs more readily phagocytosed by macrophages and induced polarization toward a pro-resolving macrophage phenotype.

rhSP-D, recombinant human surfactant protein D; rrIFN-γ, recombinant rat interferon-γ; BSYQF, Bu-Shen-Yi-Qi formula; rISM1, recombinant Isthmin-1 protein; LTC_4_, leukotriene C4; AT-RvD1, aspirin-triggered Resolvin D1; LPS, lipopolysaccharide; ATL, adenosine receptor agonist; AuNP, gold nanoparticles; GSH, reduced glutathione; hMSC, human mesenchymal stromal cells.

### 3.4 Molecular mechanisms underlying phagocytic aberrations in macrophages in asthma

Synthesis of evidence from human studies, animal investigations, and cellular research reveals that phagocytic aberrations in macrophages in asthma are not attributable to a single molecular abnormality but rather represent the cumulative outcome of alterations in the airway inflammatory microenvironment acting upon multiple molecular hierarchies. Based on the included evidence, this review categorizes the molecular mechanisms influencing macrophage phagocytic function in asthma into eight categories: dysregulation of phagocytic receptor signaling; defects at various stages of efferocytosis; immunometabolic reprogramming; aberrant signal transduction; regulation by immunomodulatory factors; acquired alterations in cellular function; circadian clock regulation; and unclassified mechanisms (**Table 5**).

**Table 5:** Synthesis of evidence for mechanistic pathways associated with phagocytic aberrations in macrophages in asthma.

Mechanistic pathway	Definition	Key molecules/processes	Study ID	Evidence sources	Consistency of evidence	Overall strength of evidence
Dysregulation of phagocytic receptor signaling	Altered expression or function of phagocytic receptors	CD36 downregulation MerTK downregulation CD64/CD11b downregulation CD206 downregulation CD64/CD11c upregulation	017, 026 030 035 026 023	Animal (1) + Cellular (2) Animal (1) + Cellular (1) Human (1) Cellular (1) Animal (1) + Cellular (1)	High High N/A N/A High	Strong Moderate Moderate Strong Moderate
Defects at various stages of efferocytosis	Impaired clearance of apoptotic cells	Impaired recognition of eat-me signals Impaired post-phagocytic signal transduction Reduced overall efferocytic efficiency	002 015, 021 012	Cellular (1) Human (1) + Animal (1) + Cellular (2) Human (1)	N/A High N/A	Moderate Strong Moderate
Immunometabolic reprogramming	Altered metabolic pathways affecting immune function	GSH/GSSG imbalance Mitochondrial dysfunction PPARδ signaling abnormality COX-2/PGE_2_ signaling abnormality	027 037 029 033	Human (1) + Cellular (1) Animal (1) + Cellular (1) Human (1) Animal (1)	High High N/A N/A	Moderate Moderate Moderate Moderate
Aberrant signal transduction	Altered intracellular signaling pathways	Arf6-mediated phagocytic cytoskeletal remodeling abnormality Rac1-mediated cytoskeletal remodeling impairment	003 009	Animal (1) + Cellular (1) Animal (1) + Cellular (1)	High High	Strong Moderate
		IRAK-M/TLR signaling pathway abnormality Leukotriene-mediated FcγR signaling enhancement Nrf2/HO-1 antioxidant pathway abnormality	007 013 022	Animal (1) + Cellular (1) Animal (1) + Cellular (1) Animal (1) + Cellular (1)	High High High	Moderate Moderate Moderate
Regulation by immunomodulatory factors	Direct regulation of phagocytic function by soluble immunomodulatory molecules	IFN-γ PGE_2_ ISM1/adiponectin	004, 008 032 011	Animal (2) + Cellular (2) Human (1) + Cellular (1) Animal (1) + Cellular (1)	High High High	Moderate Moderate Moderate
Acquired alterations in cellular function	Disease/environment-driven functional changes	Dynamic remodeling and inducible enhancement of functional activity Acquired functional deficits Inflammation-driven population turnover	006, 019, 020, 034 005, 016, 018, 024, 027, 028, 031, 036 001	Human (3) + Animal (1) + Cellular (1) Human (6) + Animal (2) + Cellular (2) Animal (1)	High High N/A	Strong Strong Moderate
Circadian clock regulation	Transcriptional regulation by circadian clock genes	Bmal1 ROR	023 010	Animal (1) + Cellular (1) Human (1) + Animal (1) + Cellular (1)	High High	Moderate Strong
Other unclassified mechanisms	Not readily classifiable into the above categories	Enhanced metabolism–phagocytosis coupling driven by pro-resolving mediators Incidental uptake dominated by physical mechanisms	014 025	Animal (1) + Cellular (1) Cellular (1)	High N/A	Moderate Moderate

Consistency of evidence was assessed based on the degree of concordance in study outcomes and mechanistic interpretations across different investigations, and categorized as high, moderate, or limited. For mechanisms addressed by only a single study, consistency was marked as not applicable (N/A). Overall strength of evidence was evaluated by comprehensively considering the number of studies, types of evidence hierarchies, study quality, consistency of findings, and macrophage source diversity, and graded as strong, moderate, or preliminary.

#### 3.4.1 Dysregulation of phagocytic receptor signaling

Macrophages rely on diverse pattern recognition receptors and phagocytic receptors to recognize, bind, and clear pathogens and apoptotic cells. Within the asthmatic pathological microenvironment, the expression or functional status of these receptors may be altered, thereby constituting an important molecular basis for phagocytic aberrations in macrophages. A total of five included studies reported changes in phagocytosis-related receptors in macrophages in asthma, involving multiple receptors including CD36, MerTK, CD64, CD11b, CD11c, and CD206. These comprised one human study, four animal studies, and five cellular studies. Overall, the consistency of evidence for this mechanistic category was high, with strong supportive evidence.

CD36 is a scavenger receptor that plays a central role in recognizing ligands such as phosphatidylserine on apoptotic cell surfaces and oxidized low-density lipoprotein; its decreased expression is closely associated with impaired phagocytic function. CD36 can be significantly downregulated under asthmatic conditions. Geiser et al. isolated airway macrophages from induced sputum of patients with mild allergic asthma and healthy controls, and found that following *in vitro* treatment with γ-tocopherol, the expression of phagocytosis-related receptors including CD36 and CD206 on macrophages from patients with asthma was significantly reduced, accompanied by suppressed expression of the co-stimulatory molecule CD86 and impaired particle internalization capacity (55). Similarly, in a BALB/c mouse model of ovalbumin (OVA)-induced asthma, CD36 expression on the surface of mouse alveolar macrophages derived from BALF exhibited a downregulated trend under asthmatic conditions, and the degree of downregulation was closely correlated with impaired phagocytic function. Mechanistically, this process may involve locally elevated tumor necrosis factor-alpha (TNF-α) in the airways promotes the cleavage and shedding of CD36 from the cell surface via activation of a disintegrin and metalloproteinase 17 (ADAM17), ultimately inhibiting macrophage recognition and uptake of apoptotic cells (24).

Mer tyrosine kinase (MerTK), an important member of the TAM receptor family, recognizes and mediates the clearance of apoptotic eosinophils, serving as a key regulatory molecule in the resolution of airway inflammation in asthma. In Mer-deficient mice (C57BL/6 background), alveolar macrophages (BALF-derived) and bone marrow-derived macrophages exhibit reduced phagocytic capacity for apoptotic eosinophils, while the bridging molecule Protein S enhances MerTK-mediated phagocytic activity. In a C57BL/6 mouse model of OVA-induced asthma, MerTK-deficient mice displayed delayed clearance of apoptotic cells, increased secondary necrosis, enhanced neutrophil recruitment, delayed resolution of eosinophilic inflammation, and exaggerated airway hyperresponsiveness, ultimately resulting in persistent airway inflammation and worsened lung function (59). Furthermore, the expression of CD64 and CD11b was downregulated in sputum macrophages from patients with mild eosinophilic asthma, and CD64 expression levels demonstrated a significant positive correlation with phagocytic function (64). Conversely, in brain and muscle ARNT-like protein 1 (Bmal1)-deficient bone marrow-derived macrophages stimulated with HDM (C57BL/6 background), CD64 and CD11c expression was upregulated, accompanied by enhanced phagocytic function (52). These studies suggest that phagocytic receptor abnormalities in asthma are not confined to a single receptor but involve coordinated changes across multiple receptors. Downregulated receptor expression, remodeling of the receptor repertoire, or restricted receptor function may each impair the efficiency of macrophage recognition, binding, and internalization of bacteria, apoptotic cells, or opsonized particles. It should be noted that under certain conditions, upregulated expression of phagocytic receptors may reflect an enhanced activation state of macrophages, but does not necessarily indicate effective initiation of inflammatory resolution programs. Therefore, dysregulation of phagocytic receptor signaling in macrophages in asthma exhibits a degree of phenotype dependence.

#### 3.4.2 Defects at various stages of efferocytosis

Efferocytosis is a critical process through which macrophages clear apoptotic cells and promote the resolution of inflammation, typically encompassing sequential stages: the release of find-me signals by apoptotic cells, macrophage recognition of eat-me signals, bridging molecule-mediated target cell binding and uptake, post-phagocytic signal transduction, and the release of anti-inflammatory and pro-resolving mediators. Impairment of efferocytosis represents one of the key mechanisms explaining the persistence of chronic inflammation in asthma. The included studies suggest that during the pathogenesis of asthma, abnormalities may occur at multiple levels, including aberrant recognition of eat-me signals, impaired post-phagocytic signal transduction, and overall reduced efferocytic efficiency. This mechanistic category involved 2 human studies, 1 animal study, and 3 cellular studies, with overall consistent evidence and strong supporting strength.

Research has demonstrated that the J774A.1 murine macrophage cell line (derived from BALB/c mice) exhibits impaired phagocytosis of apoptotic eosinophils derived from patients with asthma. Recombinant human surfactant protein D (rhSP-D) selectively induces apoptosis of eosinophils from patients with allergic asthma and, through its carbohydrate recognition domain (CRD), recognizes eat-me signals on the surface of apoptotic cells, functioning as a bridging molecule. This enhances the phagocytic clearance efficiency of apoptotic eosinophils by macrophages and promotes the resolution of allergic eosinophilic inflammation (32). It should be noted that this effect is primarily indirect, resulting from rhSP-D-induced eosinophil apoptosis and exposure of “eat-me” signals, rather than from direct activation of macrophages by rhSP-D. Nevertheless, the study directly measured macrophage uptake function using a co-culture system, thus providing direct functional evidence for macrophage efferocytosis. Furthermore, compared with patients with eosinophilic asthma, those with non-eosinophilic asthma exhibit more pronounced defects in macrophage efferocytic function, leading to inadequate clearance of apoptotic cells and persistent accumulation of neutrophils, thereby contributing to the perpetuation of chronic airway inflammation (42). Similarly, alveolar macrophages from patients with severe asthma demonstrate selective efferocytic dysfunction, accompanied by reduced secretion of the anti-inflammatory mediators prostaglandin E_2_ (PGE_2_) and 15-hydroxyeicosatetraenoic acid (15-HETE) following LPS stimulation, further impacting inflammation resolution and airway remodeling (50). Additional studies have revealed that primary alveolar macrophages from naive C57BL/6 mice, following phagocytosis of apoptotic cells *in vitro*, upregulate expression of retinaldehyde dehydrogenase 1 (RALDH1), promote regulatory T cell (Treg) generation, and suppress the production of inflammatory cytokines such as TNF-α and IL-6 under HDM stimulation, thereby attenuating airway inflammation. Furthermore, adoptive transfer of alveolar macrophages that had phagocytosed apoptotic cells into asthmatic mice significantly attenuated airway inflammatory responses (45). These findings indicate that defects in efferocytosis in asthma extend beyond the stage of apoptotic cell uptake to encompass impairment post-phagocytic immunoregulatory programs.

#### 3.4.3 Immunometabolic reprogramming

Immunometabolic reprogramming refers to the remodeling of metabolic pathways in immune cells upon activation or under stress conditions, which subsequently influences their effector functions. For macrophages, alterations in metabolic status constitute a fundamental regulatory foundation underlying phagocytic dysfunction. The included studies suggest that liver X receptor (LXR)/peroxisome proliferator-activated receptor (PPAR)-related metabolic programs, the cyclooxygenase-2 (COX-2)/PGE_2_ pathway, mitochondrial functional status, and redox imbalance may all affect the energy supply and metabolic adaptation required for macrophages to execute phagocytosis and promote inflammation resolution under asthmatic conditions. This mechanistic category involved 2 human studies, 2 animal studies, and 2 cellular studies, with overall consistent evidence and an assessed evidence strength of moderate.

Alterations in macrophage metabolic homeostasis under asthmatic conditions represent important drivers of phagocytic dysfunction. In children with severe asthma, airway oxidative stress leads to an imbalance in the glutathione/oxidized glutathione (GSH/GSSG) ratio, characterized by elevated GSSG levels and reduced histone deacetylase (HDAC) activity. This ultimately results in significantly impaired alveolar macrophage phagocytic function and increased apoptosis. Exogenous GSH supplementation reduces intracellular GSSG levels, corrects the GSH/GSSG imbalance, and thereby restores phagocytic function and reduces apoptosis in alveolar macrophages from children with severe asthma (56). Obesity-related metabolic disturbances similarly affect macrophage efferocytic function. Research indicates that obesity, through the induction of systemic metabolic abnormalities and oxidative stress, can impair peroxisome proliferator-activated receptor delta (PPARδ) signaling pathway-mediated polarization toward resolution-associated and efferocytic phenotype, thereby contributing to compromised macrophage efferocytic function in obesity-related asthma (58). Furthermore, in a BALB/c mouse model of Der f-induced asthma, macrophages undergo functional reprogramming from a pro-inflammatory state toward a pro-resolving and repair phenotype following phagocytosis of MSCs, a significant manifestation of immunometabolic remodeling, and this process may be driven by COX-2/PGE_2_ signaling (62). Another study found that human MSCs pre-treated with serum from patients with asthma develop mitochondrial dysfunction; following apoptosis, these cells are more readily phagocytosed by macrophages and further promote a pro-resolving macrophage phenotype, thereby amplifying the immunomodulatory effects of MSCs (66). Overall, a bidirectional relationship exists between macrophage phagocytic behavior and metabolic state and functional remodeling: metabolic imbalance can constrain the functional programs required for macrophages to execute uptake, processing, and inflammation resolution, while specific phagocytic events can reciprocally shape macrophage metabolism and polarization status. Consequently, even if some macrophages retain substrate uptake capacity under certain conditions, their overall phagocytic efficiency and post-phagocytic pro-resolving functions may remain impaired in the context of severe asthma or persistent inflammation, thereby contributing to the perpetuation of inflammation and disease chronicity.

#### 3.4.4 Aberrant signal transduction

The initiation and execution of macrophage phagocytosis depend on dynamic cytoskeletal rearrangement, membrane trafficking, and the finely coordinated regulation of multiple intracellular signaling pathways. Evidence from the included studies suggests that in asthma, abnormalities in various signaling molecules can not only directly impair macrophage phagocytic function but may also amplify inflammatory responses, thereby establishing a mutually reinforcing pathological cycle. This mechanistic category comprised five studies, all of which were combined animal and cellular investigations, with overall consistent findings and an assessed evidence strength of moderate.

Available evidence indicates that aberrant signal transduction, spanning multiple stages from receptor activation to cytoskeletal remodeling, constitutes an important underlying mechanism of phagocytic aberrations in macrophages. Ras-related C3 botulinum toxin substrate 1 (Rac1), a member of the Rho family of small GTPases, directly influences phagocytic cup formation and particle internalization through regulation of actin cytoskeleton reorganization, representing a critical signaling molecule in the phagocytic process. Research has demonstrated that in a BALB/c mouse model of OVA-induced asthma, Rac1 expression in peripheral blood mononuclear cells and lung tissue macrophages is downregulated, accompanied by impaired phagocytic function, accumulation of apoptotic cells, increased IL-33 release, and enhanced antigen presentation capacity, thereby promoting airway inflammation and airway hyperresponsiveness. *In vitro* experiments using the RAW264.7 murine macrophage cell line further confirmed that Rac1-mediated cytoskeletal remodeling is essential for maintaining normal phagocytic function, and diminished Rac1 expression or activity may constitute a direct cause of phagocytic function abnormality in asthma (39). In addition to Rac1, ADP-ribosylation factor 6 (Arf6) has also been implicated in asthma-related phagocytic aberrations. Arf6 serves as a critical regulatory molecule for macrophage phagocytosis of extracellular apoptosis-associated speck-like protein containing a caspase recruitment domain (ASC) specks, facilitating their uptake through modulation of actin cytoskeleton reorganization. This process limits intercellular propagation of inflammasome components and suppresses IL-1β-mediated inflammatory amplification, ultimately influencing the pathological progression of allergic asthma (41). Furthermore, IRAK-M, a negative regulator of Toll-like receptor (TLR) signaling pathways, when deficient, enhances macrophage antigen uptake capacity and activation status, accompanied by exacerbated type 2 inflammation (37). This finding suggests that enhanced phagocytic function does not necessarily equate to inflammation resolution; the biological consequences depend on the nature of the engulfed substrate and the subsequent immune programs activated. Both insufficient and aberrantly enhanced phagocytic function may, under specific circumstances, contribute to the persistence of inflammation in asthma. Additionally, certain inflammatory mediators modulate macrophage phagocytic responses through specific signaling pathways. For instance, leukotrienes generated in allergic asthma enhance alveolar macrophage phagocytic and bactericidal capacity through Fc gamma receptor (FcγR)-related pathways (43). Moreover, in OVA-induced asthmatic C57BL/6J mice, inhalation of hydrogen gas restores alveolar macrophage phagocytic function under asthmatic conditions by ameliorating oxidative stress and type 2 inflammation through repair of the impaired nuclear factor erythroid 2-related factor 2 (Nrf2)/heme oxygenase-1 (HO-1) signaling pathway (51). In summary, phagocytic aberrations in macrophages in asthma are closely associated with disturbances in cytoskeletal remodeling, imbalances in inflammatory signal transduction, and abnormalities in post-phagocytic immune programs.

#### 3.4.5 Regulation by immunomodulatory factors

The included studies suggest that various immunomodulatory factors within the asthmatic inflammatory microenvironment can directly regulate macrophage phagocytic function. This mechanistic category involves mediators such as IFN-γ, PGE_2_, Isthmin-1 (ISM1), and adiponectin, encompassing one human study, three animal studies, and four cellular studies, with relatively high consistency of evidence and strong supporting strength.

The Th1-type cytokine IFN-γ functions as a positive regulator of phagocytic function, directly enhancing macrophage phagocytic capacity and thereby attenuating airway inflammation and late-phase airway responses in asthma (34, 38). This suggests that within the predominantly type 2 inflammatory milieu of asthma, certain antagonistic immune factors may participate in inflammatory regulation through restoration of macrophage effector functions. The lipid mediator PGE_2_, in addition to its involvement in macrophage immunometabolic reprogramming via COX-2/PGE_2_ signaling, can directly inhibit macrophage phagocytosis of environmental carbon particles, contributing to phagocytic function abnormality in severe asthma (61). This indicates that the same immunomodulatory factor may exhibit distinct biological effects depending on substrate type, disease phenotype, or cellular state. Furthermore, Tee et al., through analysis of public databases, identified that reduced levels of the adipokines ISM1 and adiponectin are associated with asthma. Subsequent animal experiments (C57BL/6 mouse model of HDM-induced asthma) and cellular experiments (MH-S mouse alveolar macrophage cell line) demonstrated that ISM1 enhances the phagocytic clearance capacity of alveolar macrophages for apoptotic cells by promoting adiponectin secretion from alveolar type II epithelial cells, thereby attenuating allergic airway inflammation and airway hyperresponsiveness (41). Overall, the regulation of macrophage phagocytic function by immunomodulatory factors exhibits marked bidirectionality and dynamism. Different factors may influence macrophage clearance capacity for pathogens, particulates, and apoptotic cells through synergistic or antagonistic interactions, and across distinct asthma phenotypes, differences in the local immunomodulatory factor profile may constitute an important determinant of divergent macrophage phagocytic phenotypes.

#### 3.4.6 Other mechanisms: acquired functional alterations, circadian clock regulation, and unclassified mechanisms

Beyond the mechanisms described above, available evidence also suggests that phagocytic aberrations in macrophages in asthma may involve several mechanisms that cannot be readily categorized within the established framework, encompassing acquired alterations in cellular function, circadian clock regulation, and other unclassified mechanisms. These investigations provide important complementary insights into the molecular basis of phagocytic aberrations in macrophages in asthma.

##### 3.4.6.1 Acquired alterations in cellular function

A total of 13 studies addressed mechanisms related to acquired alterations in cellular function, spanning multiple evidence hierarchies including human, animal, and cellular investigations. These encompass diverse dimensions such as dynamic remodeling and inducible enhancement of macrophage functional activity, acquired functional deficits, and inflammation-driven cellular population turnover, with overall high consistency of evidence and strong supporting strength.

Synthesis of included studies reveals that phagocytic aberrations in macrophages in asthma do not represent static defects but rather constitute acquired functional abnormalities shaped by sustained inflammatory stimulation, oxidative stress, metabolic imbalance, and therapeutic exposures. Such aberrations may manifest either as diminished clearance capacity for pathogens or apoptotic cells, or as enhanced uptake of specific substrates accompanied by exacerbated post-phagocytic pro-inflammatory responses.

##### 3.4.6.2 Circadian clock regulation

Regulation by the circadian clock represents an emerging mechanism that has garnered increasing attention in recent years, supported by multi-level evidence from human, animal, and cellular studies. Research has demonstrated disrupted expression rhythms of circadian clock-related proteins in macrophages from patients with asthma, a finding that aligns with the characteristic diurnal variation of asthma symptoms. Core circadian molecules, including retinoic acid receptor-related orphan receptor (ROR) and brain and muscle ARNT-like protein 1 (Bmal1), regulate macrophage phagocytic function and the expression of phagocytosis-related receptors. Notably, targeting ROR receptors enhances macrophage phagocytic capacity through restoration of circadian homeostasis and promotes resolution of airway inflammation in asthma, without significantly perturbing physiological circadian rhythms (40, 52). These findings suggest that circadian disruption may not only influence symptom fluctuations in asthma but may also contribute to disease persistence through the remodeling of macrophage function.

##### 3.4.6.3 Other unclassified mechanisms

Additionally, one study revealed that alveolar macrophages from BALB/c mice with OVA-induced asthma exhibit phagocytic behavior toward nanoparticles that is primarily determined by particle size-dominated physical processes rather than by asthmatic status per se (54). This finding underscores that, in addition to the host inflammatory context, the physicochemical characteristics of the phagocytic substrate itself warrant consideration when interpreting differential macrophage phagocytic responses.

**Figure 2:**
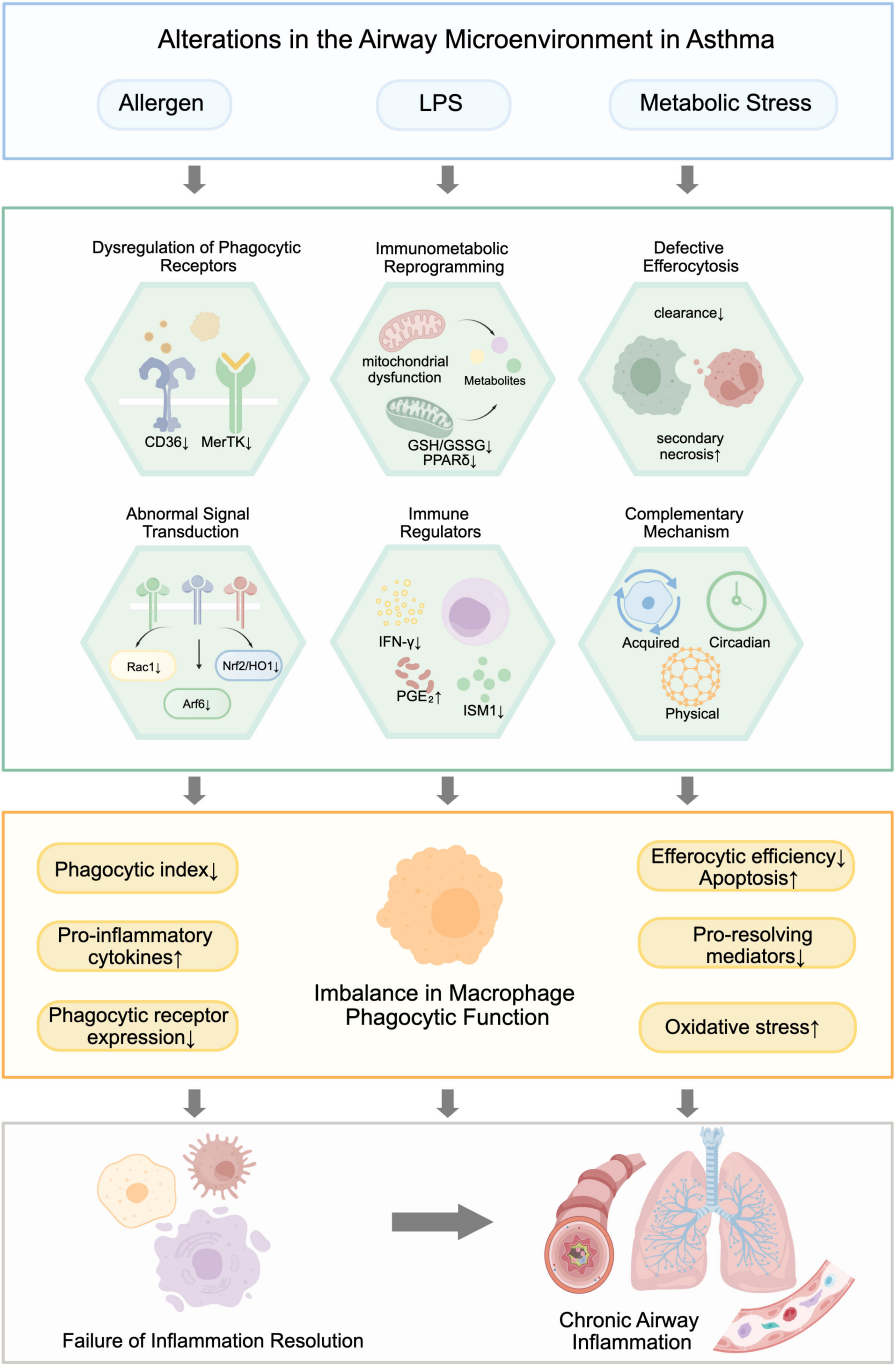
Integrative mechanisms of phagocytic aberrations in macrophages in asthma. This image, constructed based on human, animal, and *in vitro* studies included in this review, summarizes the process by which alterations in the asthmatic airway microenvironment lead to phagocytic dysregulation in macrophages through multi-layered molecular pathways. Upstream driving factors include allergen exposure, inflammatory stimuli such as lipopolysaccharide (LPS), and metabolic stress. These factors, through mechanisms including dysregulation of phagocytic receptor signaling, immunometabolic reprogramming, defects at various stages of efferocytosis, aberrant signal transduction, regulation by immunomodulatory factors, and circadian clock regulation, collectively contribute to phagocytic aberrations in macrophages, thereby resulting in failed inflammation resolution and persistent airway inflammation. Created with BioGDP.com (71).

Collectively, these mechanisms indicate that phagocytic aberrations in macrophages in asthma exhibit marked heterogeneity, dynamism, and multi-stage characteristics (**Figure 2**). The asthmatic inflammatory microenvironment drives dysregulated phagocytic receptor expression, metabolic remodeling, and aberrant signal transduction, which in turn affect phagocytic and efferocytic efficiency, ultimately resulting in diminished macrophage clearance capacity for apoptotic cells, pathogens, and particulates, or in aberrantly enhanced uptake associated with inflammatory amplification. These alterations contribute to the persistence of chronic airway inflammation, increased susceptibility to infection, elevated risk of acute exacerbations, and the development of severe or refractory asthma.

## 4 Discussion

### 4.1 Characteristics of phagocytic aberrations in macrophages supported by cross-hierarchical evidence

This systematic review integrates evidence from human studies, animal experiments, and *in vitro* investigations to systematically delineate the molecular mechanisms underlying phagocytic aberrations in macrophages in asthma. Overall, the available evidence indicates that alterations in macrophage phagocytic function constitute one of the key immunopathological features of asthma, particularly in severe asthma and specific inflammatory phenotypes. However, these alterations do not manifest as a uniform, linear decline in function but rather exhibit pronounced dependence on clinical phenotype, phagocytic substrate, and microenvironmental context.

In human studies, patients with severe asthma, non-eosinophilic asthma, and certain obesity-related asthma subtypes tend to exhibit diminished macrophage clearance capacity for pathogens or apoptotic cells, which correlates with clinical characteristics such as decreased lung function, persistent airway inflammation, and reduced glucocorticoid responsiveness. Concurrently, in some cases of mild allergic asthma or under specific exposure conditions, macrophages may demonstrate enhanced phagocytic activity, upregulated antigen uptake, or increased expression of co-stimulatory molecules. Animal and cellular studies further revealed the multi−level molecular basis of this aberration, providing a cross−hierarchical chain of evidence for understanding innate immune dysregulation in asthma.

### 4.2 Multi-pathway regulatory network within the inflammatory microenvironment

A key finding of this review is that phagocytic aberrations in macrophages in asthma do not result from a single molecule or a solitary pathway, but rather emerge as a consequence of interactions across multiple mechanistic hierarchies under the continuous remodeling influence of the inflammatory microenvironment. These mechanistic classes are not mutually exclusive but collectively constitute an interconnected and dynamically evolving regulatory network.

Dysregulation of phagocytic receptor signaling represents the most proximal molecular basis of phagocytic aberrations. Altered expression or functional status of receptors such as CD36, MerTK, can impair macrophage recognition and uptake capacity for apoptotic cells, pathogens, or particulate matter. Defects at various stages of efferocytosis, however, extend beyond simple impairment of phagocytic uptake, encompassing multiple phases from the release and recognition of find-me and eat-me signals, through bridging molecule-mediated target cell binding and internalization, to post-phagocytic immunomodulation and inflammation resolution programs. Efferocytosis normally induces anti-inflammatory and pro-resolving programs; however, in the asthmatic airway, dysfunction at any of these stages leads to accumulation of apoptotic cells. This accumulation continuously stimulates epithelial cells and immune cells, establishing a chronic inflammatory microenvironment that perpetuates inflammation in asthma, contributes to tissue damage, and affects glucocorticoid sensitivity.

Furthermore, immunometabolic reprogramming and aberrant signal transduction provide important perspectives for understanding macrophage dysfunction in severe and refractory asthma. Macrophage phagocytosis is an energy-intensive and highly programmed process requiring synergistic coordination of mitochondrial function, lipid metabolism, redox homeostasis, and cytoskeletal dynamics. Macrophage phagocytosis is an energy−intensive and highly programmed process requiring synergistic coordination of mitochondrial function, lipid metabolism, redox homeostasis, and cytoskeletal dynamics. Metabolic disturbances (e.g., GSH/GSSG imbalance, PPARδ abnormalities, and COX−2/PGE_2_ remodeling) as well as aberrations in signaling pathways can compromise the capacity of macrophages to execute phagocytic and pro−resolving responses. Thus, phagocytic aberrations in macrophages in asthma are fundamentally the result of multi-layered interactions among the inflammatory microenvironment, metabolic status, and intracellular programs.

### 4.3 Heterogeneity of phagocytic function and translational prospects of targeted modulation

Phagocytic aberrations in macrophages in asthma exhibit marked heterogeneity. Enhanced phagocytic function in mild allergic asthma may reflect stress-induced activation, whereas in severe or neutrophilic asthma, phagocytic function tends to be impaired. Therefore, macrophages in asthma exist in a state of functional reprogramming disequilibrium that should not be simplistically characterized as uniformly enhanced or diminished phagocytosis. In addition, the heterogeneity of macrophage source significantly influences their phagocytic function. During the pathogenesis of asthma, alveolar macrophages primarily exhibit impaired function, whereas monocyte-derived macrophages may display a more complex activation state. Studies using cell lines have provided important mechanistic insights into macrophage phagocytosis. However, they may not fully recapitulate the complex functional characteristics of primary cells. Therefore, future studies should prioritize functional validation using primary human alveolar macrophages and more clearly delineate distinct functional dimensions, including pathogen phagocytosis, particle uptake, efferocytosis, antigen internalization, and post-phagocytic responses, within well-defined asthma clinical phenotypes to minimize interpretive biases arising from conceptual conflation.

Building on the understanding of mechanisms underlying phagocytic aberrations in macrophages in asthma, therapeutic strategies centered on modulating phagocytic function have garnered increasing attention in recent years (67, 68). Existing studies have identified several promising directions for asthma intervention. For example, exogenous GSH supplementation, administration of rhSP-D, application of aspirin-triggered resolvin D1 (AT-RvD1), ellagic acid, and MSC-based interventions have demonstrated potential for improving macrophage phagocytic or efferocytic capacity across different levels of evidence. However, the heterogeneity of asthma phenotypes constitutes a critical determinant of the success of targeted intervention. For patients with severe asthma and those with non-eosinophilic asthma characterized by persistent neutrophilic accumulation, restoration of phagocytic and efferocytic capacity may be particularly beneficial in promoting the resolution of airway inflammation and reducing the risk of acute exacerbations. In contrast, for patients with mild allergic asthma, enhancing phagocytic function could potentially exacerbate antigen presentation and amplify inflammatory responses. Consequently, future therapeutic strategies targeting macrophage phagocytic function should emphasize phenotype matching, functional stratification, and directionally specific restoration; that is, selecting differential intervention pathways that promote clearance, limit pro-inflammatory activation, or reconstitute inflammation resolution programs based on the specific pattern of macrophage functional disequilibrium characteristic of each asthma phenotype.

### 4.4 Limitations of available evidence and future research directions

This systematic review was conducted and reported in accordance with the PRISMA guidelines, with prospective registration, ensuring rigorous screening, review, and categorization of the 37 included studies. Furthermore, by simultaneously incorporating human, animal, and *in vitro* studies, this review provides a comprehensive integrative analysis across three levels, namely clinical phenomena, *in vivo* validation, and molecular mechanisms, relatively systematically presenting a continuous chain of evidence for phagocytic aberrations in macrophages in asthma. Additionally, this study not only focuses on macrophage phagocytic function per se but also synthesizes distinct mechanistic pathways and their interconnections, thereby constructing a more comprehensive mechanistic framework.

This review also has several limitations. First, the included studies exhibited substantial heterogeneity in study populations, definitions of asthma phenotypes, macrophage sources, detection methods, and outcome measures, which precluded quantitative meta-analysis and constrained direct comparisons across studies. Second, human studies generally had small sample sizes, lacking large-scale investigations, and exhibited inadequate control for potential confounding variables such as ICS use, smoking status, age, and obesity across different studies. Finally, animal studies exhibited an overall high risk of bias, primarily involving selection bias, performance bias, detection bias, and reporting bias, which to some extent limits the strength of causal inference. Although some cellular studies provided mechanistic evidence, they still lack sufficient validation using human samples or support from *in vivo* causal evidence.

Future efforts should strengthen stratification of asthma clinical phenotypes and establish more standardized assessment frameworks for phagocytic function to enhance comparability across studies. Further integration of single-cell transcriptomics, spatial transcriptomics, metabolomics, and functional experiments is warranted to construct a more comprehensive mechanistic atlas of macrophage phagocytic function in asthma. Recent transcriptomic studies have characterized the gene expression profiles of airway macrophages in asthma (69, 70). However, direct evidence linking these transcriptional signatures to macrophage phagocytic function remains lacking. Therefore, integrating multi-omics analyses with functional phagocytosis assays represents a crucial direction for future research to elucidate the relationship between asthma phenotypes and macrophage phagocytic function. Additionally, more high-quality prospective clinical studies are needed to validate the predictive value of phagocytic function-related parameters as biomarkers in asthma and to explore whether intervention strategies targeting macrophage phagocytic function can ameliorate airway inflammation, reduce the risk of acute exacerbations, and improve long-term clinical outcomes in patients with asthma.

## 5 Conclusion

This systematic review demonstrates that alterations in macrophage phagocytic function in asthma do not represent a unidirectional functional impairment but rather a state of functional aberration characterized by phenotype dependence, substrate dependence, and microenvironmental dependence. Synthesis of evidence from human, animal, and *in vitro* studies reveals that dysregulation of phagocytic receptor signaling, defects at various stages of efferocytosis, immunometabolic reprogramming, aberrant signal transduction, regulation by immunomodulatory factors, acquired alterations in cellular function, and circadian clock regulation collectively constitute a multi-layered molecular regulatory network underlying phagocytic aberrations in macrophages in asthma. The systematic integration of these mechanisms not only expands the understanding of innate immune dysfunction in asthma but also suggests that macrophage phagocytic function may represent a critical entry point for asthma phenotyping, disease severity assessment, and targeted intervention. Future research necessitates higher-quality, standardized studies incorporating cross-hierarchical validation designs to further characterize phagocytic aberrations in macrophages across distinct asthma phenotypes and their amenability to intervention, thereby facilitating the translation of these mechanisms into clinical practice and providing novel therapeutic avenues for severe and refractory asthma.

## Data Availability

The original contributions presented in the study are included in the article/supplementary material. Further inquiries can be directed to the corresponding author.

## Glossary

ICS: Inhaled corticosteroids
Th2: Type 2 T helper
IL: Interleukin
AMs: Alveolar macrophages
DAMPs: Damage-associated molecular patterns
PRISMA: Preferred Reporting Items for Systematic Reviews and Meta-Analyses
MeSH: Medical Subject Headings
NOS: Newcastle-Ottawa Scale
BALF: Bronchoalveolar lavage fluid
LPS: Lipopolysaccharide
OVA: Ovalbumin
HDM: House dust mite
IFN-γ: Interferon-gamma
MSCs: Mesenchymal stem cells
RSV: Respiratory syncytial virus
IRAK-M: Interleukin-1 receptor-associated kinase M
MFI: Mean fluorescence intensity
TNF-α: Tumor necrosis factor-alpha
ADAM17: A disintegrin and metalloproteinase 17
MerTK: Mer tyrosine kinase
rhSP-D: Recombinant human surfactant protein D
CRD: carbohydrate recognition domain
PGE₂: Prostaglandin E₂
15-HETE: 15-hydroxyeicosatetraenoic acid
RALDH1: Retinaldehyde dehydrogenase 1
Treg: Regulatory T cell
LXR: Liver X receptor
PPAR: Peroxisome proliferator-activated receptor
COX-2: Cyclooxygenase-2
GSH: Glutathione
GSSG: Oxidized glutathione
HDAC: Histone deacetylase
PPARδ: Peroxisome proliferator-activated receptor delta
Rac1: Ras-related C3 botulinum toxin substrate 1
Arf6: ADP-ribosylation factor 6
ASC: apoptosis-associated speck-like protein containing a caspase recruitment domain
TLR: Toll-like receptor
FcγR: Fc gamma receptor
Nrf2: Nuclear factor erythroid 2-related factor 2
HO-1: Heme oxygenase-1
ISM1: Isthmin-1
Bmal1: Brain and muscle ARNT-like protein 1
ROR: Retinoic acid receptor-related orphan receptor
AT-RvD1: Aspirin-triggered resolvin D1
